# Mitogenomes of Eight Nymphalidae Butterfly Species and Reconstructed Phylogeny of Nymphalidae (Nymphalidae: Lepidoptera)

**DOI:** 10.3390/genes14051018

**Published:** 2023-04-29

**Authors:** Zhen-Tian Yan, Zhen-Huai Fan, Shu-Lin He, Xue-Qian Wang, Bin Chen, Si-Te Luo

**Affiliations:** 1 Chongqing Key Laboratory of Vector Insects, Institute of Entomology and Molecular Biology, College of Life Sciences, Chongqing Normal University, Chongqing 401331, China; 2 School of Life Sciences, Xiamen University, Xiamen 361102, China

**Keywords:** Nymphalidae, mitochondrial genome, phylogenetic analysis

## Abstract

The Nymphalidae family of cosmopolitan butterflies (Lepidoptera) comprises approximately 7200 species found on all continents and in all habitats. However, debate persists regarding the phylogenetic relationships within this family. In this study, we assembled and annotated eight mitogenomes of Nymphalidae, constituting the first report of complete mitogenomes for this family. Comparative analysis of 105 mitochondrial genomes revealed that the gene compositions and orders were identical to the ancestral insect mitogenome, except for *Callerebia polyphemus trnV* being before *trnL* and *Limenitis homeyeri* having two *trnL* genes. The results regarding length variation, AT bias, and codon usage were consistent with previous reports on butterfly mitogenomes. Our analysis indicated that the subfamilies Limenitinae, Nymphalinae, Apaturinae, Satyrinae, Charaxinae, Heliconiinae, and Danainae are monophyletic, while the subfamily the subfamily Cyrestinae is polyphyletic. Danainae is the base of the phylogenetic tree. At the tribe level, Euthaliini in Limenitinae; Melitaeini and Kallimini in Nymphalinae; Pseudergolini in Cyrestinae; Mycalesini, Coenonymphini, Ypthimini, Satyrini, and Melanitini in Satyrinae; and Charaxini in Charaxinae are regarded as monophyletic groups. However, the tribe Lethini in Satyrinae is paraphyletic, while the tribes Limenitini and Neptini in Limenitinae, Nymphalini and Hypolimni in Nymphalinae, and Danaini and Euploeini in Danainae are polyphyletic. This study is the first to report the gene features and phylogenetic relationships of the Nymphalidae family based on mitogenome analysis, providing a foundation for future studies of population genetics and phylogenetic relationships within this family.

## 1. Introduction

The Nymphalidae family is a sizable group within the Lepidoptera order, with over 7200 species existing globally, and it is classified into 13 subfamilies [1]. The butterflies belonging to the Nymphalidae family exhibit a diverse array of colors and striking forms. [2]. Studies conducted on Nymphalidae have yielded invaluable insights into ecological genetics, behavioral science, biodiversity, and conservation biology [3,4,5,6]. In recent decades, Nymphalidae has become an important model group in the fields of ecology, evolutionary biology, population genetics, and conservation biology due to its rich species diversity, morphological diversity, and unique biogeographic distribution [7,8]. The classification system of the 13 subfamilies developed by Ackery is now widely accepted, which includes Heliconiinae, Nymphalinae, Limelitinae, Charaxinae, Apaturinae, Satyrinae, Morphinae, Calinaginae, Danainae, Cyrestidinae, Pseudergolinae, Libytheinae, and Biblidinae [9]. Furthermore, the ten-subfamily classification system presented in *The Butterfly Records of China* by Chou Io is widely accepted within China; it encompasses the following subfamilies: Charaxinae, Heliconiinae, Apaturinae, Pseudergolinae, Argynninae, Limelitinae, Byblinae, Marpesiinae, Nymphalinae, and Calinaginae [2,10]. Currently, the phylogenetic relationships within the Nymphalidae family remain incompletely resolved, and certain research results suggest that the relationship between Nymphalidae and other families may be structured as follows: (((Nymphalidae + Lycaenidae) + Pieridae) + Papilionidae) [11,12]. However, Chai et al. reconstructed the phylogenetic tree of butterflies by utilizing complete sequences of mitochondrial genomes (mitogenomes) and discovered that Nymphalidae and (Lycaenidae + Pieridae) were sister groups. The phylogenetic relationship among families was identified as (((Lycaenidae + Pieridae) + Nymphalidae) + Papilionidae) [13]. As a consequence of inconsistencies within previous classifications of Nymphalidae, we sequenced the mitogenomes of eight Nymphalidae butterflies to explore their phylogeny. Despite this endeavor, the phylogenetic and monophyletic relationships between the subfamilies remain undefined, and the phylogenetic positions of some subfamilies in other molecular studies contrast. Comprehensive research will be necessary to resolve these issues. The mitochondrion is a crucial organelle found in nearly all eukaryotic organisms, and it plays a vital role in energy metabolism, aging, apoptosis, disease regulation, and fatty acid and specific proteins’ synthesis [14,15]. The mitochondrion serves as the central hub for converting and transferring energy through oxidative phosphorylation, providing indispensable energy to the cell [14]. Mitochondrial genomes are circular, double-stranded molecules of approximately 15~20 kb in size [14].

Insect mitochondrial DNA is distinguished by its simple structure, rapid evolution speed, high copy number, and ease of separation and purification. These properties render it an outstanding molecular marker for studying insect systematics [16,17]. The size of mitochondrial DNA in butterflies is typically around 14~16 kb, which includes 37 protein-coding genes (PCGs), 22 transfer RNA genes, 2 ribosomal RNA genes (large ribosomal RNA, rrnL; small ribosomal RNA, rrnS), and 1 A+T-rich region. The varying length of the A+T-rich region in the mitochondrial genome results in distinct mitochondrial lengths. This region carries multiple regulatory elements that play essential roles in replication and transcription; hence, it is known as the mitochondrial DNA control region [18,19]. Presently, the commonly employed genes for molecular systematic studies on butterflies include *16S rDNA*, *Cytb*, *COI*, *COII*, and *ND5. 16S rDNA* and *Cytb* are generally more conserved, while *COI*, *COII*, and *ND5* evolve at a faster rate [20,21]. As different mtDNA gene sequences have variable mutation rates, there are various genes suitable for analyzing the phylogenetic relationships of different taxonomic orders [22]. Compared with a complete mitochondrial genome, individual genes can only reflect some information and provide limited effective information quantity, while entire mitochondrial genome sequences are more abundant and can provide information on all genes, including non-coding regions, and all relevant genetic information, such as tRNA and ribosomal RNA secondary structures and other advanced structural information. Numerous research results currently available in the literature, which were obtained as a result of determining the gene arrangement of the mitochondrial genome, different pathways of genetic evolution, other forms of potential information, and the entire genome itself, contain aspects that can be used as a molecular evolution research object; therefore, complete mitochondrial genome sequences are the most powerful forms of insect molecular phylogenetic research evidence [23,24]. For this study, we sequenced and annotated the entire mitochondrial genomes of eight Nymphalidae species. In total, 107 mitochondrial genomes were utilized to construct 2 phylogenetic trees, consisting of 8 new mitogenomes, 97 previously published mitochondrial genomes of Nymphalidae, and 2 outgroups from Papilionidae. To better comprehend the functions of related genes, we analyzed the relative synonymous codon usage (RSCU) and AT skew values of protein-coding genes and compared them to those of other mitogenome sequences in Nymphalidae. Through our findings, we have gained innovative insights into the phylogenetic relationships of numerous significant subfamilies and tribes within Nymphalidae.

## 2. Materials and Methods

### 2.1. Sample Collection, Identification, Sequencing, and Mitogenome Assembly

All of the specimens used in this study were collected from China and preserved at Chongqing Normal University (No. 20190816006; contact: Zhentian YAN: 20132148@cqnu.edu.cn). The samples used in this study were collected and returned to the laboratory for wing spreading and identification, which were mainly based on the Illustrated Handbook of Chinese Butterflies and the Classification and Identification of Chinese Butterflies [10,25]. Genomic DNA extraction was carried out using the TIANamp Genomic DNA Kit (TIANGEN, Beijing, China). The Illumina Truseq™ DNA Sample Preparation Kit (Illumina, San Diego, CA, USA) was utilized to produce the sequencing library, which was conducted according to the manufacturer’s instructions. The constructed library was then loaded onto an Illumina Novaseq 6000 platform for PE 2 × 150 bp sequencing, which was performed by Novogene (Beijing, China). Quality control of the raw genomic data was performed using FastQC v0.11.5 software (http://www.bioinformatics.babraham.ac.uk/projects/fastqc, accessed on 1 March 2023). Fastp v0.23.2 (https://github.com/OpenGene/fastp, accessed on 1 March 2023) was used for quality trimming and filtering of the data, wherein reads containing over 5% unknown nucleotides or more than 50% bases with Q-values ≤ 20 as well as unpaired reads were discarded. The filtered data were utilized to assemble the complete mitochondrial genome using GetOrganelle v1.7.6.1, with ‘animal_mt’ in the default database serving as the seed reads [26]. Mitoz v3.4 was utilized to perform the annotation of the complete mitogenomes [27].

### 2.2. Mitogenome Annotation and Characteristics Analysis

The Mitos tool (http://mitos.bioinf.uni-leipzig.de/index.py, accessed on 1 March 2023) was utilized to identify the preliminary boundaries of each gene [28]. By conducting comparisons with homologous sequences of mitogenome sequences of Nymphalidae in GenBank, all 13 protein-coding genes (PCGs) and 2 rRNA genes were determined. Geneious v.4.8.5 was utilized to curate the preliminary sequence results [29].The MAFFT v6. 06 tool was employed to identify tRNA, [30]. The graphical maps of the mitogenomes were visualized using the CGView Comparison Tool [31]. MEGA v.6.06 software was used to calculate the amino acid content, base composition, and relative synonymous codon usage (RSCU) [32]. To explore the nucleotide composition bias, AT-skew [(A − T)/(A + T)] and GC-skew [(G − C)/(G + C)] were calculated [33]. The software DNASP v.5.10.01 was used to calculate the synonymous mutation rates (Ks), non-synonymous mutation rates (Ka), and the ratio of Ka to Ks (Ka/Ks) in 13 protein-coding genes (13PCGs) [34]. A box plot was created using OriginPro v.2019b to examine the Ka/Ks ratio for each gene [35].

### 2.3. Phylogenetic Analysis

Phylogenetic relationships among 105 Nymphalidae species, consisting of 8 species from this study and 97 known species, were inferred using one mitochondrial genome database (PCG123+two rRNAs—protein-coding genes with all codon positions—12S rRNA and 16S rRNA) and two methods (BI—Bayesian inference and ML—Maximum likelihood). *Teinopalpus aureus* (Lepidoptera: Papilionidae) and *Paranticopsis xenocles* (Lepidoptera: Papilionidae) sequences were used as outgroups, and their collection information and accession numbers are presented in Table S3. The PCG123 dataset was created using SequenceMatrix, and multiple sequence alignments of 13 PCG genes were accomplished using MAFFT v6.06 [36,37]. Ambiguous regions were removed using Gblocks. Phylogenetic analyses were conducted using PhyloSuite v1.2.2 (http://dongzhang0725.github.io/, accessed on 1 March 2023) [38]. The optimal partition schemes and nucleotide substitution models were determined using PartitionFinder 2.0, based on Bayesian information criterion (BIC) [39,40]. MrBayes 3.2.6 was implemented for BI analysis, employing 2,000,000 generations and 4 chains and sampling every 1000 generations. A consensus tree was constructed after discarding the first 25% of trees as burn-in, and posterior probabilities (PPs) were calculated. The best-fit mode was calculated using the Akaike information criterion (AIC) in ModelFinder. Subsequently, the maximum-likelihood (ML) phylogenetic tree was reconstructed using IQ-TREE (v 2.1.2), with 1000 ultrafast bootstraps, according to the GTR+F+R6 model [41].

## 3. Results

### 3.1. Genome Organization and Nucleotide Composition

**Figure 1:**
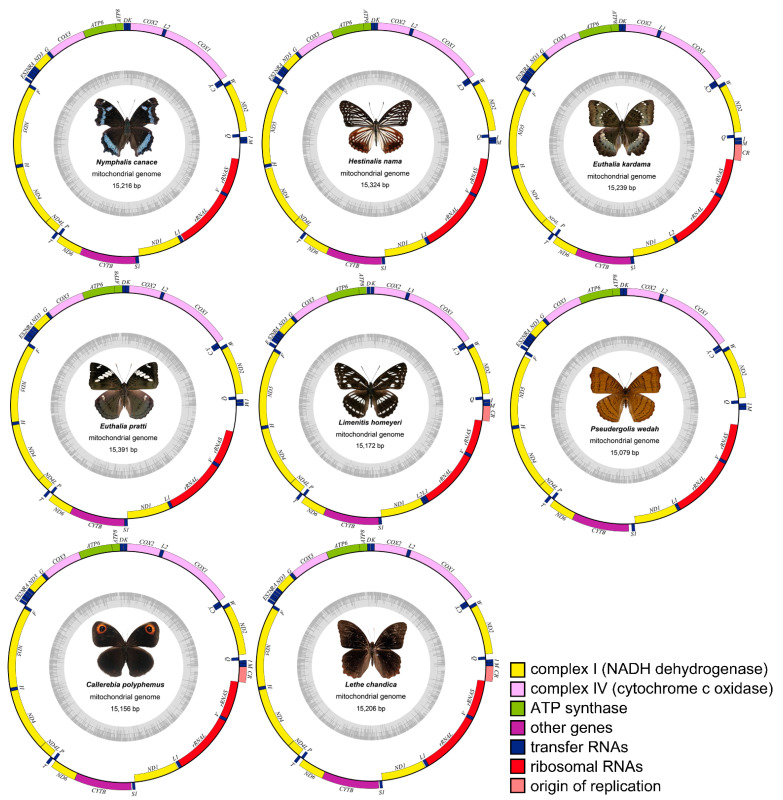
Complete mitogenomic structures of Nymphalis canace, Hestinalis nama, Euthalia kaedama, Euthalia pratti, Limenitis homeyeri, Pseudergolis wedah, Callerebia polyphemus, and Lethe chandica. The genes on the outer loop are on the J-strand, and the genes on the inner loop are on the N-strand. L1, L2, S1, and S2 represent tRNA-Leu(UAA), tRNA-Leu(UAG), tRNA-Ser(UGA), and tRNA-Ser(GCU), respectively.

This study reports the first complete mitochondrial genome sequences of eight Nymphalidae species (Table 1)—all of which were previously unreported. The lengths of the complete mitochondrial genomes in Nymphalidae range from 14,957 bp (*Junonia iphita*) to 15,615 bp (*Bhagadatta austenia*), for which variations mainly attributed to the Conserved Region (CR) length and overlaps and gaps. All 105 mitogenomes (8 new and 97 known) exhibit no genetic rearrangements and encode 37 genes, including 13 PCGs, 22 tRNAs, 2 rRNAs, and a CR, except for *L. homeyeri*, which has two *trnL* genes. In the annotated mitogenomes, fourteen tRNAs and nine PCGs are located on the majority strand (J-strand), whereas eight tRNAs, four PCGs, and two rRNAs are located on the minority strand (N-strand), except for *H. nama*, which has fourteen tRNAs and nine PCGs on the majority strand (N-strand) and seven tRNAs, four PCGs, and two rRNAs on the minority strand (J-strand) (Figure 1). There are 18 to 23 intergenic spacers and 1 to 5 overlapping regions between genes in the complete mitochondrial genome of the 8 species studied. The total intergenic spacer length ranges from 87 bp (*C. polyphemus*) to 276 bp (*H. nama*), with the longest intergenic spacer detected between *tRNA^Gln^* and *ND2* in *H. nama*. Additionally, there are overlapping regions of 7 bp (*L. chandica*) to 50 bp (*H. nama*) in various genes, with the longest overlapping region being 19 bp between *tRNA^Try^* and *COI* in *H. nama* (Table S1). The mitochondrial genomes of all 105 species show an evident AT bias, with AT content ranging from 79.05% (*Melanargia asiatica*) to 81.95% (*Athyma sulpitia*). The AT-skew, which characterizes the nucleotide composition bias, is almost balanced and ranges from −0.0673 (*C. biblis*) to 0.0205 (*P. wedah*).

### 3.2. Characteristics of Mitogenomes Genes

**Figure 2:**
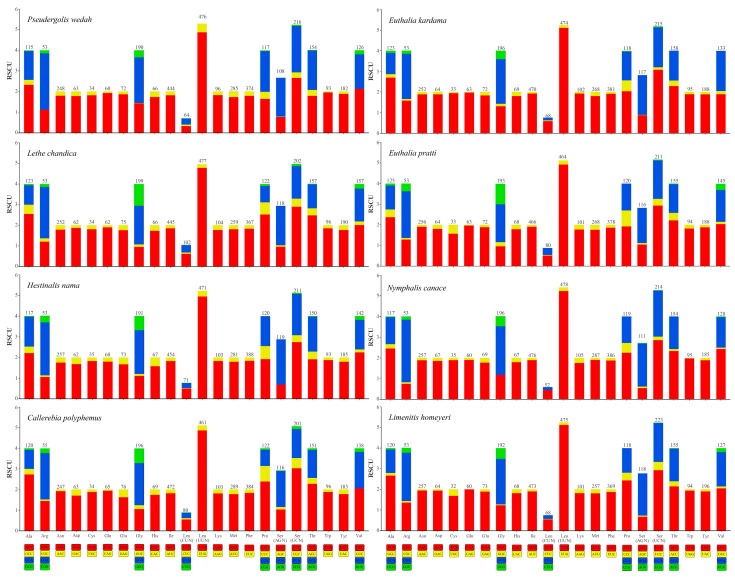
Relative synonymous codon usage (RSCU) of 13 PCGs in the mitogenomes of the eight newly sequenced species.

**Figure 3:**
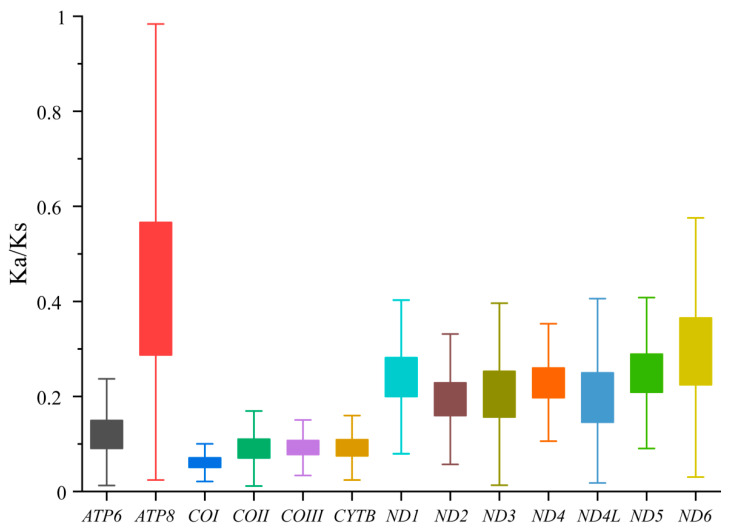
Box plot of Ka/Ks from 13 PCGs of 105 Nymphalidae mitogenomes.

Among the 13 protein-coding genes (PCGs), the lengths range from 10,947 bp (*P. wedah*) to 11,258 bp (*B. austenia*), with AT content ranging from 80.7% (*A. sulpitia*) to 76.9% (*H. autonoe*) (Table S2). Most of the start codons in the 13 PCGs are ATN, but some specific codons, such as TTA, CTA, and GTA, are present in *ND5*, *ND4*, *ND4L*, and *ND1*. The most common stop codons are TAA and TAG, while the rarest stop codon is AAT, and the partial codon T is also present. Regarding codon usage, UUU (Phe), UUA (Leu), and AUU (Ile) are the most common, while UAG (*), ACG (Thr), and CUG (Leu) are used the least. Codons ending with A/U (92.61%) are more commonly present as opposed to those ending with G/C (7.39%) (Figure 2). The Ka/Ks ratios of the 13 PCGs are all lower than 0.5, with the highest Ka value occurring in *ATP8* (0.171) and the lowest in *COI* (0.049). Among the genes, *COIII* has the highest Ks value (0.436), while *ND5* has the lowest Ks value (0.269). The Ka/Ks ratio of *COI* is the lowest (0.124), while that of *ATP8* is the highest (0.438) (Figure 3).

Like other Nymphalidae species, the mitogenomes of these eight species were found to have twenty-two transfer RNA genes. Among the 22 transfer RNAs (tRNAs), the lengths range from 58 bp (trnV) to 74 bp (trnL). All these tRNAs form conventional clover-leaf structures, except tRNA-Ser (AGN), which lacks a dihydrouridine (DHU) arm [42].

The average lengths of the two ribosomal RNAs are estimated to be 1339 bp (rrnL) and 792 bp (rrnS), with A + T content values of 84.5% (rrnL) and 85.2% (rrnS), respectively.

The control region (CR), located between trnM and rrnS, varies in length from 311 bp (*C. polyphemus*) to 565 bp (*E. pratti*), with A + T content ranging from 79.4% (*C. polyphemus*) to 95.2% (*E. pratti*). The nucleotide compositional behavior of mtgenomes has been widely measured using three parameters: AT-skew, GC-skew, and A + T content (A + T%). The research conducted with respect to these eight species indicates that the AT-skew of three of them (*H. nama, K. canace,* and *P. wedah*) was slightly lower than the average AT-skew of Nymphalidae (−0.1541). Additionally, the GC-skew of three of the species (*Euthalia kardama, Euthalia pratti,* and *Nymphalis canace*) was slightly higher than the average AT-skew of Nymphalidae (0.0201). The A + T content and GC content indicate a consistent pattern, with Nymphalidae mtgenomes having a higher percentage of A + T. 

### 3.3. Phylogenetic Relationships

**Figure 4:**
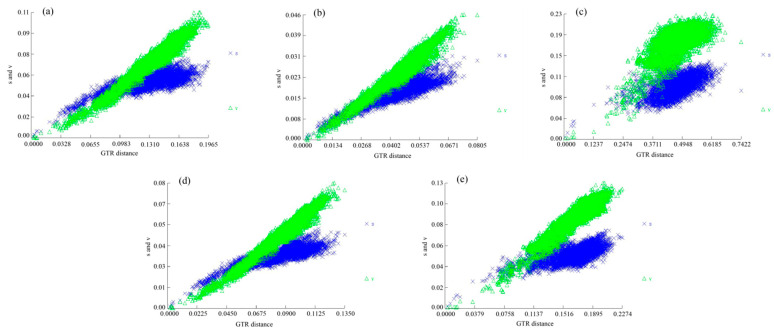
Substitution saturation test results, based on PCG1 (**a**), PCG2 (**b**), PCG3 (**c**), PCG12 (**d**), and PCG123 (**e**). The scatter is distributed as y = x, indicating that it is not saturated and can continue to build trees. The blue (×s) for transitions and the green (Δv) for transversion.

The nucleotide substitution saturation test indicated that none of the five nucleotide sequence datasets, namely, PCG1, PCG2, PCG3, PCG12, and PCG123, exhibit saturations (all Iss values are less than Iss.cSym or Iss.cAsym, and *p*-values are less than 0.05). This conclusion is supported by the accompanying graphs [43,44] (Figure 4 and Table 2).

**Figure 5:**
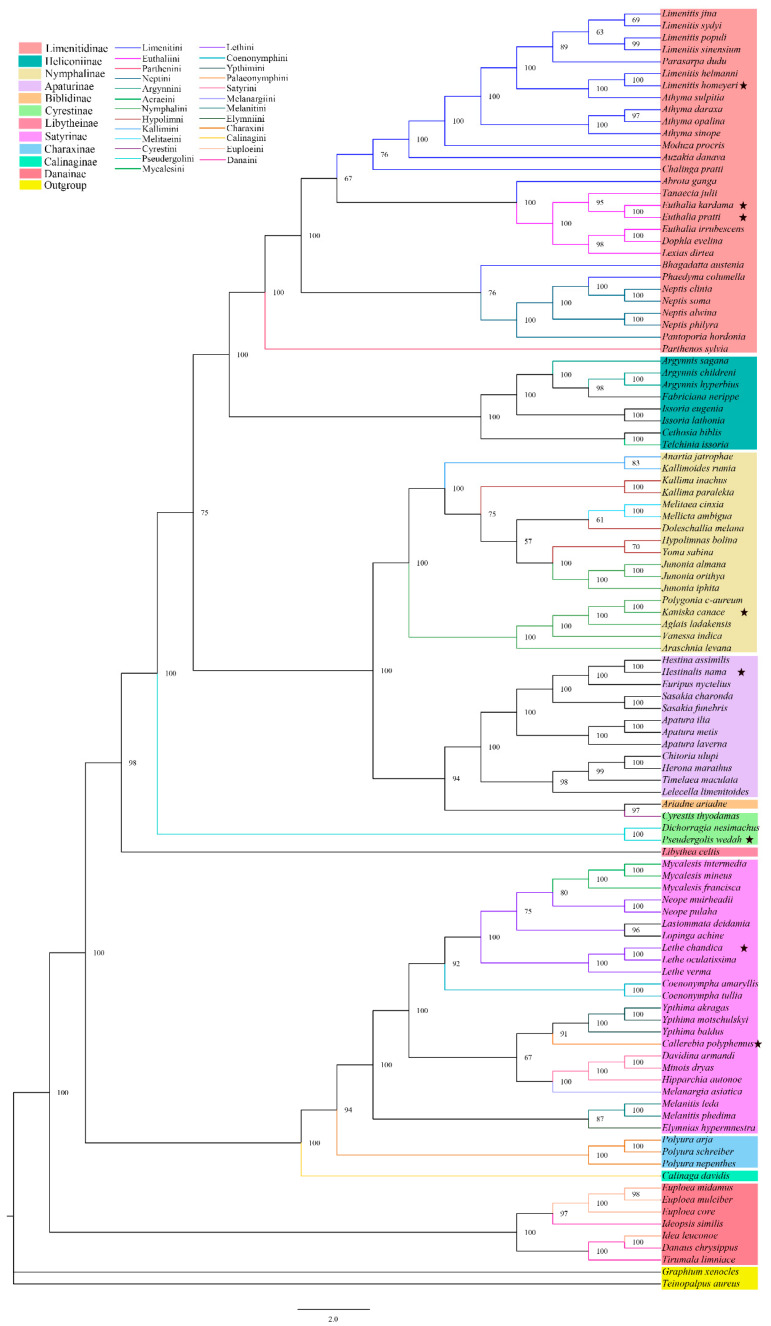
Reconstruction of a phylogenetic tree determined via Maximum likelihood method based on PCGs+two rRNAs of 107 butterfly mitogenomes. Bootstrap support values are indicated in branches. Stars (⋆) indicate the newly sequenced species.

**Figure 6:**
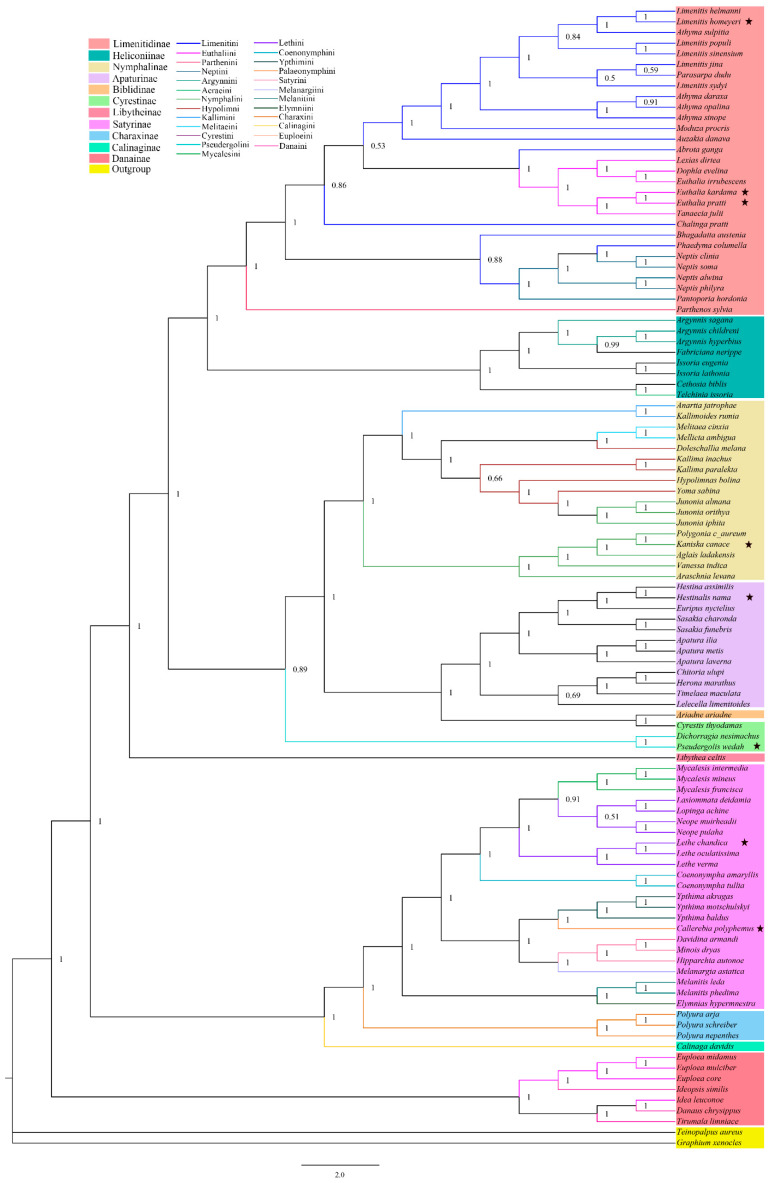
Reconstruction of a phylogenetic tree determined via Bayesian inference methods based on PCG123+two rRNAs of 107 butterfly mitogenomes. Bayesian posterior probabilities (BPP) are shown at relevant branches of the BI tree. Stars (⋆) indicate the newly sequenced species.

We utilized the PCG123 and two rRNA datasets to construct Bayesian inference (BI) and Maximum likelihood (ML) phylogenetic trees, which we then analyzed to investigate the phylogeny of Nymphalidae. Our results suggest that Danainae (PP = 1; BP = 100), Charaxinae (PP = 1; BP ≥ 94), Satyrinae (PP = 1; BP ≥ 94), Apaturinae (PP = 1; BP ≥ 94), Nymphalinae (PP = 1; BP = 100), Heliconiinae (PP = 1; BP = 100), and Limenitinae (PP = 1; BP = 100) are monophyletic subfamilies, while also suggesting that Cyrestinae is polyphyletic. Among the subfamilies, Danainae was found to be the base of the phylogenetic trees, followed by the subfamily Calinaginae in which there was only one species included in the analysis. We also found that Limenitidinae and Heliconiinae as well as Satyrinae and Charaxinae were sister groups. (Figure 5 and Figure 6). Furthermore, our analysis addressed the controversial question of which subfamily is the base of Nymphalidae, with the result suggesting that Danainae assumed this position. However, further supporting evidence is still required (See Figure 5 for details).

At the tribe level, we found that Calinaginae contained only one species, *C. davidis*, within the tribe Calinagini. Satyrinae included ten tribes, with Elymniini, Palaeonymphini, and Melanargiini each containing only one species (*Elymnias hypermnestra, Callerebia polyphemus*, and *Melanargia asiatica*, respectively). In Charaxini (PP = 1; BP ≥ 94), we analyzed three species, while Melanitini (PP = 1; BP ≥ 87), Ypthimini (PP = 1; BP ≥ 91), and Satyrini (PP = 1; BP = 100) each had three species. Coenonymphini (PP = 1; BP ≥ 92) and Mycalesini (PP ≥ 0.91; BP ≥ 80) were determined to have two and three species, respectively. These six tribes were determined to be monophyletic. The last tribe, Lethini, included seven species, but they were separated into three clades, resulting in a paraphyletic group.

The subfamily Cyrestinae is comprised of two tribes: Pseudergolini (PP ≥ 0.89; BP = 100) and Cyrestini (with only one species, *Cyrestis thyodamas*). Within Nymphalinae, we identified four tribes that contained a total of seventeen species. Kallimini (PP = 1; BP = 100) and Melitaeini (PP = 1; BP ≥ 61) each had two species and were determined to be monophyletic. Hypolimni was divided into three clades, while Nymphalini was found to be polyphyletic and divided into two clades. In Heliconiinae, it was suggested that Argynnini was paraphyletic (Figure 5 and Figure 6).

Lastly, we examined Limenitinae, which consists of four tribes. Parthenini contained only one species, while two species (*Phaedyma columella* and *Bhagadatta austenia*) were linked to the tribe Neptini. Euthaliini (PP = 1; BP = 100) was positioned between the two parts of Limenitini, and we identified Euthaliini as monophyletic but Neptini as paraphyletic, while Limenitini was determined to be polyphyletic.

## 4. Discussion

### 4.1. General Characteristics

The present study has sequenced and annotated the mitogenomes of eight Nymphalidae species for the first time, all of which are the first to have complete mitogenomes reported. These mitogenomes contain 37 genes and a CR, which exhibit a clear AT bias. The gene compositions and sequences of these mitogenomes remain the same as their ancestors. The mitogenome lengths range from 14,957 bp (*J. iphita*) to 15,615 bp (*B. austenia*), where the length variation mainly occurs in the CR. Among the 13 PCGs of the eight species under study, most of the initial codons corresponded to ATN (N = A, T, C, and G). However, there are some relatively infrequent start codons, such as CTA and TTA in *ND5* as well as TTA and GTA in *ND4, DN4L*, and *ND1.* The most frequent termination codon is TAA, and an incomplete stop codon T also exists in the complete sequences of other Lepidoptera mitochondrial genomes [45,46,47]. Our study has identified some gene spacing and overlap in the sequences, which could potentially provide valuable phylogenetic information. However, further research is necessary to expand the scope and include more species in order to draw more conclusive results. Our study has revealed a unique occurrence of two *tRNA-Leu* genes *(uag)* in *L. homeyeri,* which are not present in other Nymphalidae species. Such gene duplication events have been closely linked to the evolution of genome size in living organisms, the emergence of new genes, species differentiation, and the capacity of genes to withstand mutations [48]. Nevertheless, the underlying reasons and impacts of this phenomenon require further investigation in order to obtain a clearer understanding.

### 4.2. Subfamily-Level Phylogenetic Relationships in Nymphalidae

The results of this study indicate that the subfamilies Limenitinae, Nymphalinae, Apaturinae, Satyrinae, Charaxinae, Heliconiinae, and Danainae demonstrate monophyletic characteristics, while the subfamily Cyrestinae shows polyphyletic features. Based on our findings, it appears that the Danainae subfamily is the most primordial in terms of evolution and constituting the basis of the phylogenetic trees. The Danainae subfamily forms sister groups with the other subfamilies in Nymphalidae. A prior study that analyzed *COI, EF-1α*, and wingless genes from 54 species found that the Libytheinae subfamily constitutes a distinct basal branch within Nymphalidae and is a sister group to the other subfamilies [49]. The present study has identified four distinct evolutionary clades within Nymphalidae: the Danaine, Satyrine, Heliconiine, and Nymphaline clades. Specifically, the Danaine clade includes Danainae; the Satyrine clade encompasses Charaxinae, Satyrinae, Calinaginae, and Morphinae; the Heliconiine clade encompasses Heliconiinae and Limenitinae; and the Nymphaline clade encompasses Nymphalinae, Apaturinae, Cyrestinae, Biblidinae, and Pseudergolinae. A phylogenetic analysis, based on both morphology and molecular biology, which included 10 genes and 235 characters from 400 species representing all major lineages of the Nymphalidae family, identified Libytheinae as the basal branch of Nymphalidae. Furthermore, the study suggested that Libytheinae and Danaidae constitute two separate branches of the family [50]. This study categorized the Nymphalidae family into five distinct evolutionary clades, which included the Danaine clade, the Satyrine clade, the Heliconiine clade, the Nymphaline clade, and the Libytheine clade. Specifically, the Satyrine clade comprises Satyrinae, Charaxinae, and Calinaginae; the Nymphaline clade encompasses Nymphalinae, Apaturinae, Cyrestinae, Biblidinae, and Pseudergolinae; the Heliconiine clade includes Heliconiinae and Limenitinae; and the Libytheinae and Danainae form two distinct clades. Our Bayesian Inference analyses revealed that the Danaine + (Satyrine + (Libytheine + (Nymphaline + Heliconline))) clade was retrieved, which verified previous mitogenomic studies that focused on Nymphalidae [51,52,53]. On the contrary, an earlier study reported that Danainae, instead of Libytheinae, serves as the basis for the Nymphalidae family and forms a sister group with the other subfamilies within the Nymphalidae [11]. A study based on the mitogenomes of *Euploea mulciber* and *Libythea celtis* suggested that Libytheinae may not be the basis of Nymphalidae but rather that Danainae is the basis of Nymphalidae, forming sister groups with other subfamilies of Nymphalidae [54]. Although the first two findings are somewhat divergent from our current research, the last two conclusions align with our outcomes [11,49,50]. These variations in results could be attributed to a range of factors, such as differences in the sequencing samples, sequencing methods, selected genes, or tree-building methodologies, and many more. Though there have been some recent advancements in the phylogenetic studies of the Nymphalidae and several evidential materials have been proposed for such analyses, most of these studies use either mitochondrial genes or nuclear genes as molecular markers. To fully address the ongoing disputes surrounding Nymphalidae, further research may be required to explore additional molecular markers or methods, including techniques from other related disciplines such as morphology and molecular biology, to complement traditional methodologies.

### 4.3. Tribal-Level Phylogenetic Relationships in Nymphalidae

The present study suggests that several tribes within certain subfamilies of the Nymphalidae family are categorized as distinct monophyletic groups. Specifically, Euthaliini in Limenitinae; Melitaeini and Kallimini in Nymphalinae; Pseudergolini in Cyrestinae; Mycalesini, Coenonymphini, Ypthimini, Satyrini, and Melanitini in Satyrinae; and Charaxini in Charaxinae were found to belong to monophyletic groups. On the other hand, the study identified several tribes that exhibit polyphyletic features, such as Danaini and Euploeini in Danainae; Limenitini in Limenitinae; Nymphalini and Hypolimni in Nymphalinae; and Lethini in Satyrinae. To date, a holistic and structured examination of the molecular systematics of the Limenitinae subfamily remains limited. Prior research conducted on the basis of *COI* and *Cytb* genes, however, suggested that Euthaliini and Neptini were monophyletic groups [55,56,57,58]. For this investigation, a total of 3090 base pairs of DNA sequences were analyzed from both the mitochondrial gene *COI* and the nuclear genes *EF-1α* and *wingless* for 165 Satyrinae specimens. As a result, the findings indicate that Melanitini is considered monophyletic, whereas the Satyrinae subfamily is categorized as polyphyletic based on our analysis [59]. Based on morphology, several scholars have suggested that the subtribe Heliconiiti constitutes a monophyletic group [60]. One study constructed NJ, ML, and MP trees relying on the *COI* gene of 19 species to analyze the evolutionary relationships within the Nymphalidae family. The results revealed that both Junoniini and Nymphalini have monophyletic characteristics, while Kallimini was not categorized as a monophyletic group [61]. In a 2021 study concerning Limenitidinae, Maximum Likelihood analysis revealed a relationship with a structure of (Parthenini + ((Chalingini + (Cymothoini + Neptini)) + (Adoliadini + Limenitidini))) and Bayesian analysis based on the same dataset revealed a relationship with the structure (Parthenini + (Adoliadini + ((Cymothoini + Neptini) + (Chalingini + Limenitidini)))) [62]. Our research shows that the relationship within the Limenitidinae subfamily is structured as follows: (Parthenini+ (Neptini + (Limenitini + Euthaliini))). This result is similar to the result obtained using BI in 2021. Comparing these findings with previous research, we discovered that the group relationships of the tribes Neptini in Limenitinae and Kallimini in Nymphalinae demonstrated some variation from previous conclusions. Given the limited number of studies conducted on Nymphalidae tribes, further evidence and reliable references will be essential for future examination. The reasons for these differences in phylogenetic inferences could be due to various factors, such as the inclusion or exclusion of certain groups and differences in methodologies and data selection. Consequently, there is a need for further investigation at the tribe level in order to better comprehend and establish the evolutionary relationships within the Nymphalidae family.

## 5. Conclusions

This study provides a detailed analysis of the correlation between the mitochondrial genome and the phylogeny of Nymphalidae. Comparative analysis revealed similarities in terms of genetic composition and sequences between species and their ancestors, such as identical AT skew, codon utilization rate, and variation length in insects. In addition, the overlap and variation between genes presented important phylogenetic information. On the other hand, repetition and conservation in the CR did not contribute as much. The subfamilies Limenitinae, Nymphalinae, Apaturinae, Satyrinae, Charaxinae, Heliconiinae, and Danainae were observed to be monophyletic, whereas the subfamily Cyrestinae was determined to be polyphyletic. The study revealed that Danainae is the base subfamily in the analyzed phylogenetic trees. At the tribe level, various monophyletic groups were identified, such as Euthaliini, Melitaeini, and Kallimini; Pseudergolini, Mycalesini, Coenonymphini, Ypthimini, Satyrini, and Melanitini; and Charaxini. However, the tribe Lethini was found to be paraphyletic, and the tribes Limenitini and Neptini, Nymphalini and Hypolimni, and Euploeini were regarded as polyphyletic. The phylogenetic analysis confirmed the placement of the eight species within the Nymphalidae family.

This study utilized a larger set of sequences and more diverse analytical perspectives compared to other related studies, resulting in a more convincing reconstruction of Nymphalidae’s phylogenetic relationships. However, there were a few shortcomings in this study, including the exclusion of some butterfly mitochondrial genomes that were not of optimal quality. Future studies should aim to enhance the available mitochondrial genome resources of butterflies for more comprehensive and enriched research on this family.

## Figures and Tables

**Table 1:** Taxonomic and collection information of the eight newly sequenced species in Nymphalidae.

No.	Subfamily	Species	Accession No.	Collected Location
1	Nymphalinae	*Kaniska canace* Linnaeus, 1763	MZ481931	Zhaomu Mountain Forest Park, Chongqing (106.514° E, 29.633° N)
2	Apaturinae	*Hestina nama* Douleday, 1844	MZ501810	Love ladder, Jiangjin District, Chongqing (106.335° E, 28.728° N)
3	Limenitinae	*Euthalia kardama* Moore, 1859	MZ501803	Jinfo Mountain, Chongqing (107.134° E, 29.054° N)
4		*Euthalia pratti* Leech, 1891	MZ501809	Jinfo Mountain, Chongqing (107.134° E, 29.054° N)
5		*Limenitis homeyeri* Tancre,1881	MZ501806	Wangxiangtai, Simian Mountain, Jiangjin District, Chongqing (106.45° E, 28.679° N)
6	Cyrestinae	*Pseudergolis wedah* Collar, 1844	MZ501808	Nanshan Botanical Garden, Chongqing (106.635° E, 29.561° N)
7	Satyrinae	*Callerebia polyphemus* Oberthür, 1877	MZ491831	Jinfo Mountain, Chongqing (107.134° E, 29.054° N)
8		*Lethe chandica* Moore, 1858	MZ501804	Nanshan Botanical Garden, Chongqing (106.635° E, 29.561° N)

**Table 2:** Substitution saturation test results.

Data Partition	Iss	Iss.cSym ^†^	Psym ^‡^	Iss.cAsym ^§^	Pasym ^¶^
PCG123	0.224	0.857	0	0.846	0
PCG12	0.126	0.853	0	0.845	0
PCG1	0.172	0.849	0	0.836	0
PCG2	0.077	0.849	0	0.836	0
PCG3	0.489	0.849	0	0.836	0

^†^ Index of substitution saturation assuming a symmetrical true tree; ^‡^ probability of significant difference between Iss and Iss.cSym (two-tailed test); ^§^ index of substitution saturation assuming an asymmetrical true tree; ^¶^ probability of significant difference between Iss and Iss.cAsym (determined via two-tailed test).

## Data Availability

The genome sequence data that support the findings of this study are openly available in GenBank of NCBI at (https://www.ncbi.nlm.nih.gov/) under accession no OP499856 on 26 March 2023. The associated BioProject, SRA, and Bio-Sample numbers are PRJNA948885, PRJNA948884, PRJNA948882, PRJNA948881, PRJNA948880, PRJNA948877, PRJNA948878, PRJNA948874, SRR23973998, SRR23973997, SRR23973996, SRR23973995, SRR23973994, SRR23973992, SRR23973993, SRR23973991, SAMN33921810, SAMN33921808, SAMN33921806, SAMN33921777, SAMN33921775, SAMN33921773, SAMN33921774, and SAMN33921772.

## Supplementary Materials

The following supporting information can be downloaded at: https://www.mdpi.com/article/10.3390/genes14051018/s1. Table S1: Basic sequence characteristics of mitochondrial genomes of eight newly sequenced species in Nymphalidae; Table S2: Base composition, AT content, AT-skew, GC-skew, and total length (bp) of 105 species in all three codon positions, namely, codon position 1, codon position 2, and codon position 3, investigated in this study. The species highlighted in red are the ones that were sequenced; Table S3: Taxonomic information and GenBank accession numbers for 105 mitogenomes in Nymphalidae selected for characteristics and phylogenetic analysis in this study (“/” represent species that do not yet have a clear classification at the tribe level).
